# Adolescents’ Perspectives on the Barriers and Facilitators of Physical Activity: An Updated Systematic Review of Qualitative Studies

**DOI:** 10.3390/ijerph18094954

**Published:** 2021-05-06

**Authors:** João Martins, João Costa, Hugo Sarmento, Adilson Marques, Cláudio Farias, Marcos Onofre, Miguel González Valeiro

**Affiliations:** 1 Centro de Estudos de Educação, Faculdade de Motricidade Humana e UIDEF, Instituto de Educação, Universidade de Lisboa, 1649-004 Lisboa, Portugal; jmartins@fmh.ulisboa.pt (J.M.); monofre@fmh.ulisboa.pt (M.O.); 2 Facultad de Ciencias del Deporte y la Educación Física, Universidad de A Coruña, 15001 A Coruña, Spain; maglez@udc.es; 3 ISAMB, University of Lisbon, 1649-004 Lisboa, Portugal; 4 CIPER, Faculty of Human Kinetics, University of Lisbon, 1649-004 Lisboa, Portugal; 5 Sports Studies and Physical Education Programme, School of Education, University College Cork, T12 YN60 Cork, Ireland; joao.costa@ucc.ie; 6 Research Unit for Sport and Physical Activity (CIDAF), Faculty of Sport Sciences and Physical Education, Estádio Universitário de Coimbra, University of Coimbra, 3040-256 Coimbra, Portugal; hugo.sarmento@uc.pt; 7 Faculty of Sport, University of Porto, Rua Doutor Plácido Costa, 91, 4200-450 Porto, Portugal; claudiofarias@fade.up.pt; 8 Centre of Research, Education, Innovation, and Intervention in Sport (CIFI2D), Faculty of Sport, University of Porto, Rua Doutor Plácido Costa, 91, 4200-450 Porto, Portugal

**Keywords:** exercise, sport, physical education, youth, correlates, young people’s voices, qualitative synthesis

## Abstract

Listening to adolescents’ voices has been important to promote meaningful physical activity (PA) opportunities. Therefore, an updated systematic review of the available qualitative literature on adolescents’ perspectives on the barriers and facilitators of PA was conducted, according to Preferred Reporting Items for Systematic Reviews and Meta-Analyses guidelines. Studies published between 2014 (date of the last systematic review) and 2020 were searched in the Web of Science, EBSCO, and SCOPUS databases. Based on the inclusion criteria applied, 30 out of 8069 studies were included in the review. A thematic analysis was used to inductively and deductively analyze the perspectives of ~1250 adolescents (13–18 years). The studies took place in 13 countries from different continents. The main PA barriers and facilitators of PA were presented and discussed around five higher-order themes: (1) Individual factors (e.g., psychological—motivation, self—efficacy; cognitive—knowledge, understanding; physical—motor skills); (2) social and relational factors (family, friends, significant others); (3) PA nature factors (fun, school-based PA and physical education); (4) life factors (time and competing activities; life-course); and (5) sociocultural and environmental factors (e.g., availability/access to PA facilities, programs; urban/rural zones). By transnationally framing adolescents’ voices, this study provides updated evidence and discusses innovative implications for developing tailored interventions and pedagogical strategies aimed at promoting active and healthy lifestyles.

## 1. Introduction

Recently, the World Health Organization (WHO) updated the guidelines on physical activity (PA). Children and adolescents should do at least an average of 60 min per day of PA with moderate-to-vigorous intensity, mostly aerobic, across the week. In addition, vigorous intensity aerobic activities, as well as those that strengthen muscle and bone, should be incorporated at least 3 days a week [1]. However, research on children and adolescents’ PA shows that the overall PA levels tend to be low, decline with age, and are particularly lower in girls and low socioeconomic status (SES) groups [2,3,4]. As such, it is essential to continue the research on the factors that contribute or inhibit adolescents to engage with PA in its different forms, to minimize their sedentary lifestyle and reap all the PA benefits that will contribute to a fuller, longer, better, and happier life.

PA can be conceptualized as a complex behavior, influenced by diverse multilevel factors that can interact across contextual levels from the individual to the macro-system [5]. Given the complex and multifactorial relationships influencing the adolescents’ PA participation [6,7,8], research needs to adopt ecologically relevant frameworks to capture and inform on individual, social, and contextual factors that enable higher and better PA levels among those ages. Building on the ecological paradigm, and particularly on Bronfenbrenner’s ecology of human development framework [9], socio-ecological models (SEM) in PA [5] have gained relevance for their potential to understand PA behaviors and inform relevant interventions for promoting PA [10].

A substantive amount of research on facilitating or hindering factors of PA participation relies on quantitative approaches through correlates and determinants [6,7,11]. However, qualitative studies addressing the adolescents’ voices are paramount to fully capture the extent and depth of this complex behavior, and its interdependence within and across the different levels and contexts [12,13,14]. By combining findings of such qualitative studies in reviews, research can get a comprehensive overview of essential elements that inform and update further large-scale quantitative studies and scalable interventions. Such increase in explanatory power is essential as previous systematic reviews on the interventions’ effectiveness document limited evidence in increasing PA in key groups [15] and a very limited impact on overall PA [16]. Additionally, the focus on young people’s voices has been considered to be crucial since this might help further understand how the barriers and facilitators might be shaped by individual, social and environmental contexts [13,17], and therefore, to better inform meaningful PA intervention strategies in diverse contexts, such as in school, physical education (PE), sport clubs, active recreation, and active travel [5,18,19,20].

Under this view, recent research has been engaging with the efforts to summarize the qualitative findings of youth PA, helping to establish barriers and facilitators of PA in specific youth demographics [12,13,14,17,21]. In brief, some of the main commonly PA barriers identified across these reviews were related to: Lack of fun, motivation, and perception of competence; body image and gender bias in sport and PA; lack of support from family, friends, and significant others—such as coaches and PE teachers; negative experiences in PA and PE contexts; competition and highly structured PA opportunities; and limited environmental opportunities. Conversely, the main PA facilitators suggested by young people in these reviews were: Positive PA attitude; fun, motivation and perception of competence; perception of body image and challenging stereotypes; friends, family, and significant others support for PA—such as PE teachers; positive experiences in PE and PA; a safe environment; and access to PA programs and recreational infrastructures. Nevertheless, these reviews have focused on specific youth demographics such as UK [12,14], only girls [17], children with disability [21] or from urban contexts and high-income economy countries [13]. 

As such, a wider and more comprehensive review is needed to update this body of knowledge and further understand the facilitators and barriers of PA from the perspective of adolescents with different characteristics (e.g., sex, PA levels and trajectories, urban/rural contexts, country income). Moreover, Martins et al. [13] discussed that most studies did not report the SES of participants and that a clear picture of PA was not provided, calling on detailing SES, and including mixed methods study designs involving a qualitative dimension. Thus, this study updates previous systematic reviews on the qualitative nature of PA barriers and facilitators, as well as broadens the contextual spectrum by not limiting the analysis to urban contexts and high economy countries.

## 2. Materials and Methods

An updated systematic review [13] of the available qualitative literature on adolescents’ perspectives on the barriers and facilitators of PA was conducted according to Preferred Reporting Items for Systematic Reviews and Meta-Analyses Guidelines [22]. Similar inclusion criteria and search strategy followed by Martins et al. [13] were adopted in the current study.

### 2.1. Inclusion and Exclusion Criteria

The predefined eligibility criteria for including the scientific articles in the present review allowed updating and extending the previous systematic literature review [13]. The criteria were as follows: (1) Studies that explored the perspectives of adolescents on PA, as well as on PA facilitators and barriers and that directly reported adolescents’ perspectives/voices regarding those issues (outcome criteria); (2) empirical studies that were observational and that used qualitative methods to gather data (design criteria); (3) adolescents aged between 13 and 18 years, healthy, and from urban and non-urban areas living in developed or developing countries (population criteria); (4) studies published in English, French, Portuguese or Spanish (language criteria); (5) studies published between 2014 (date of the last systematic review [13] on this specific subject) and 6 June 2020 (time criteria); and (6) articles published in scientific journals (publish criteria). 

Studies were excluded if they: (1) Were not focused on exploring the perspectives of adolescents on PA facilitators or barriers; (2) did not directly report the ‘voice’ of adolescents; (3) had an experimental design or were a review; (4) did not use qualitative methods; (5) did not involve participants aged 13–18 years old; (6) did involve participants with diverse age ranges but the mean age did not belong to the 13–18 years old age bracket or the data for the diverse age groups was not presented separately for this specific age group; (7) were focused on participants with non-healthy conditions (e.g., obese; mental health issues) or physical disabilities; (8) were not published in English, French, Portuguese or Spanish; (9) were not published between 2014–2020; (10) were not articles published in scientific journals with peer review (e.g., conference papers); and (11) scored low for both reliability and usefulness in the study quality evaluation based on evidence for policy and practice information (EPPI) criteria [23,24] (detailed below).

### 2.2. Search Strategy

As for the search strategy, ‘Web of Science’, ‘EBSCO’, and ‘SCOPUS’ databases were used to ensure, from an early stage, the scientific quality of the studies. The search strategy was based on the following fields ‘title’, ‘abstract’, and ‘keywords/subject’. The language of publication was restricted to English, Spanish, Portuguese, and French. The terms used in the search were: ‘adolescen*’ OR ‘young people’ OR ‘youth’ OR ‘teen*’ OR ‘student*’ AND ‘physical activity’ OR ‘physical education’ OR ‘exercise’ OR ‘sport*’ OR ‘active’ OR ‘inactive’ AND ‘correlate*’ OR ‘determinant*’ OR ‘facilitator*’ OR ‘barrier*’ OR ‘factor influen*’ OR ‘socio-ecological factors’ OR ‘psychosocial factor*’ OR ‘environmental factor*’ AND ‘qualitative’ OR ‘mixed-method*’ OR ‘focus group*’ OR ‘interview*’ OR ‘narrative*’ OR ‘discourse*’ OR ‘view*’ OR ‘perspective*’ OR ‘voice*’ OR ‘experience*’ OR ‘grounded theory’. Additional records were identified through reference lists. In those cases where the scientific article or data needed were not available (e.g., no access to pdf; no mean age), the authors of the study were contacted via email and/or professional media platforms.

### 2.3. Studies Screening, Selection, and Quality

After performing the search in the databases, the data was imported into a reference manager software (EndNote X9, 2013, Clarivate Analytics, Philadelphia, PA, USA). Duplicates were eliminated automatically. Two authors screened titles and abstracts of the remaining records. Each full-text article was independently examined by the first author (J.M.) and a second author (H.S. or J.C.) to decide whether the article met the inclusion criteria and if so, to assess its quality. Disagreements among reviewers were solved by consensus.

The EPPI criteria [23,24] were used to assess the quality of the articles and, consequently, the risk of bias. In the first phase, the EPPI criteria included the analysis of the following six indicators: [1] Were steps taken to increase rigor in the sampling?; [2] were steps taken to increase rigor in the data collected?; [3] were steps taken to increase rigor in the analysis of the data?; [4] were the findings of the study grounded in/supported by the data?; [5] rating of the findings of the study in terms of their breadth and depth; and [6] to what extent does the study privilege the perspectives and experiences of children?. In a second phase, based on the scores of previous indicators, each study was rated in terms of their [7] reliability and [8] usefulness of its findings for the present review, by using the following scale: Low, medium, and high. Considering the rating of each study by two independent researchers (J.M.; J.C., C.F. or H.S.), no study was excluded based on the quality threshold of scoring low for both reliability and usefulness.

### 2.4. Data Extraction, Analysis, and Synthesis

Initially, each article was read, and the following characteristics were extracted independently by the first author of the present review and by another author: (1) First author’s name and publication year; (2) aim; (3) theoretical framework; (4) sample; (5) data collection and analysis procedures; and (6) results’ themes. Data extracted by two researchers were reexamined together, readjusted, and confirmed. Next, with the support of the MAXQDA 2020 software (Verbi Software, Berlin, Germany) [25], a thematic synthesis approach [26] was adopted to analyze and synthesize the data concerning the main PA facilitators and barriers according to adolescents’ voices and perspectives. In this inductive/deductive process, each article was read several times and analyzed line by line by the first author of this study. Next, the main PA barriers and facilitators of the articles were inductively identified (Supplementary Table S1), constantly compared, and then coded according to the thematic emphasis in sub-themes and higher-order themes. This process of the thematic synthesis was also performed by taking into consideration the socio-ecological model of health behavior [5] and the key barriers and facilitators of PA identified in the previous systematic review [13]. As such, the socio-ecological model provided guidance for sub-themes, whereas the higher-order themes were generated to highlight important relationships throughout the socio-ecological model dimensions as framed by Sallis and Owen [5]. Adopting a socio-ecological lens is important since it can provide multi-layered connected lenses to understand the importance of individual, social, environmental, and political factors related to PA behavior [5,27]. The thematic synthesis was an inductive/deductive and iterative process led by the first author and involving the co-authors (e.g., for categorical system refinement, reexamining, and confirming themes).

## 3. Results

### 3.1. Study Selection

**Figure 1:**
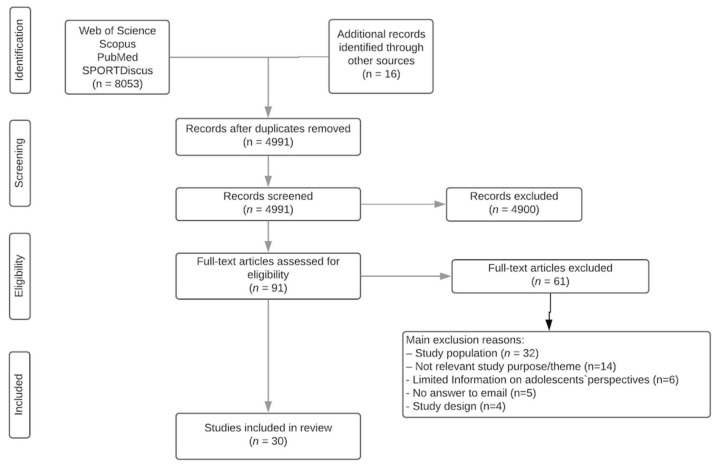
PRISMA flow diagram for study selection.

The initial search identified 8053 records in the described databases, and an additional 16 records were identified through the reference list. These data were then exported to the reference manager software EndNote X9 [28] and all duplicates (3078 records) were eliminated automatically. The remaining 4991 studies were then screened according to the title and abstract for relevance, resulting in another 4900 studies being eliminated from the database. The full text of the remaining 91 studies was read and another 61 were rejected due to a lack of relevance for the specific purpose of the current review. The main reason for exclusion was related to the study population ages (not 13–18 years old) (*n* = 32). Other reasons for exclusion are identified in Figure 1. At the end of the screening procedure, 30 studies received further in-depth reading, their quality was evaluated and, as a consequence, all were included in the review.

### 3.2. Study Characteristics

Table 1 presents the characteristics of the studies between 2014 and 2020. For the present review, the voices and perspectives of ~1250 adolescents from countries around the world were taken into account. Ten studies were performed in the UK, four in Spain, three in the USA, two in Portugal, and two in Iran, as well as one in each one of the following countries: Belgium, Estonia, Netherlands, Canada and Colombia, Australia, India, Morocco and South Africa. Considering the World Bank Classification (2014–2020, https://www.worldbank.org (accessed on 2 December 2020) the majority of countries were from a high-income economy, two were from an upper-middle-income economy (South Africa, Iran), and two from a lower-middle-income economy (Morocco and India).

In Table 1, it is also possible to identify that 26 studies were cross-sectional (23 qualitative, three mixed methods) and four studies had a longitudinal design. Most studies combined two technics of data collection (mainly questionnaire and interview).

As for PA, 14 out of 30 studies did not measure the adolescents’ PA levels. PA was self-reported in a questionnaire and/or in an interview in 14 other studies. PA was also identified based on the PE teacher’s subjective classification [29] or by recurring to accelerometry [30]. Overall, the majority of studies collecting the adolescent’s PA levels, have involved and considered in the analysis the perspectives of (i) active adolescents [31,32,33]; (ii) adolescents who have been active for the last 8 years [34]; (iii) adolescents with different levels of PA [27,29,30,35,36,37]; and (iv) inactive or low active adolescents [38,39,40]. Two studies involved adolescents from diverse PA levels but did not stratify the results based on the identified PA levels [41,42].

As for additional characteristics of the participants, 10 studies focused exclusively on girls. About one-third of the papers did not report the SES of the participants. For those which did, SES was often reported at a general level and not used in the analysis. Some studies, however, involved only adolescents with a low SES [37,43] or explicitly contrasted the voices of adolescents with a low and a high SES [27,36,44]. 

About 50% of the studies mentioned the participants’ ethnicity, with some focusing only on participants from one specific ethnicity, such as Latin [39] or South Asian Muslim girls [45,46,47,48]. Of the 30 studies, two studies focused purposively on adolescents from rural zones [29,43], two from urban zones [33,40], and two explored the perspectives of urban vs. rural adolescents [44,49].

As for the study quality, based on the previously explained evidence for policy and practice information (EPPI) [23,24] criteria, in Table 1 it is possible to identify that: 11 studies were classified with medium reliability and usefulness; five studies with high reliability and medium usefulness; five studies with medium reliability and high usefulness; and nine studies with both high reliability and usefulness.

Regarding each study purpose (Supplementary Table S2), most studies focused on exploring the perspectives of adolescents about the main PA facilitators and barriers. However, some studies also had a particular focus on the socio-cultural discourses about the body [35], femininity [31], and power relations [29]; on knowledge and understanding of PA and health [42]; on specific contexts such as school-based PA and PE e.g., [36,37,50] or public open spaces [33]; on the social support of friends or family e.g., [27,30,32]); on the recommendations to promote PA e.g., [40,51,52]; and on the understanding of the factors related to the PA decline with age [38]. The socio-ecological model of health promotion was the theoretical framework mostly used in seven studies. Hill Collins’ matrix of domination and intersectionality appears in four studies, all from the same author [45,46,47,48]. Thirteen studies have not reported the use of any theoretical model.

### 3.3. Results of Individual Studies and Synthesis of Principal PA Facilitators and Barriers

The main PA barriers and facilitators of the analyzed studies were inductively and deductively identified and presented for each study (Supplementary Table S1). A total of five higher-order themes and 14 sub-themes that represent the adolescents’ perspectives on the main PA facilitators and barriers are systematized in Table 2 and presented below. For the purpose of presenting the findings, the 14 sub-themes will be focused as the perceived barriers and facilitators, whereas the five higher-order themes will structure the discussion of these findings, since they facilitate to explore the interactions across ecological levels as framed by Sallis and Owen [5].

#### 3.3.1. Theme 1: Individual Factors

##### Physical and Motor Skills

Low physical fitness, exhaustion, tiredness, injuries, being overweight or obese, having health problems, physical discomfort, and limited motor skills were identified as important PA barriers by adolescents in 14 studies. Conversely, having good physical fitness and sport skills were mentioned as PA facilitators by active adolescents [27,35,36,49].

##### Physical Activity Attitude, Knowledge, and Understanding

A negative attitude towards PA emerged as an important barrier for adolescents with different characteristics, but mainly with low PA levels, across 21 studies. For those adolescents, PA was not often appreciated, valued, and was not part of their self-identity e.g., [29,41,47,52,53,54]. A negative PA attitude became more prevalent with age [31,37,43,54] and was often associated with negative PA experiences. These adolescents did not like activities that were: imposed, repetitive, boring, traditional [31,45,46,53,54]; highly structured, intense, and non-challenging [29,40]; focused on performance/competition e.g., [34,35,36,38,48], and team sports [39,40,45,49]; where no autonomy/choice was given [53,54,55]; and that occurred in a non-supportive environment e.g., [29,31,51,52]. Contrariwise, a positive attitude towards PA emerged as an important facilitator in the discourses of active adolescents from 22 studies. Those adolescents were mainly physically active, passionate about PA, and an active lifestyle was considered to be part of their self-identity e.g., [34,35,36,56]. They preferred and recommended activities that were: competitive e.g., [35,36,51,53,56]—often by boys and girls with higher perceived competence and sport skills—and non-competitive [37,52]; informal/unstructured and inclusive [33,40,52]; challengingly appropriate e.g., [29,36,51]; new, diversified, adventurous, and fun [36,45,53,54]; characterized by a game element [51]; meaningful and transferable to different life contexts [40,49]; where interaction with friends is possible [29,30,31,32,36,54]; that occur in a supportive and mastery-oriented learning environment [31,37,51,52]; and where adolescents are given a voice e.g., [27,31,34,42,45,52,54,55]. 

Regarding the knowledge and understanding, several PA benefits related to physical and mental health, body image, weight management, fitness, academic performance, socialization, sleep, and general life skills were identified by adolescents in several studies e.g., [35,42,43,44,48,49,55,56], but mainly by those who tended to be physically active. Interestingly, some adolescents also talked about their ability to self-regulate their learning and PA behavior, recurring to the internet/apps as well as to transfer and apply their knowledge to different PA contexts e.g., [34,55]. Nevertheless, several issues that may prevent PA participation emerged in the adolescents’ voices regarding their limited knowledge and understanding of the: PA, health, and fitness concepts e.g., [37,43,44,55,56]; PA recommendations for health e.g., [36,37,42,56]; psychosocial PA benefits [37,42,44]; and planning ability for being physically active [42,55].

##### Motivation

Lack of motivation or extrinsic motivation (e.g., lose weight to avoid humiliation) was a major PA barrier indicated, mainly by inactive adolescents, across 15 studies e.g., [38,43,55,56,57]. Instead, being intrinsically motivated emerged as a PA facilitator in the discourses of active adolescents from 14 studies e.g., [31,32,34,53]. In the adolescents’ discourses, it was possible to identify that progression in learning and performance, having fun, positive interpersonal relationships, autonomy, self-efficacy, and self-regulatory skills favored motivation e.g., [37,52,54,55], as in the case of adolescents that were active for at least 8 years [34].

##### Perception of Competence and Self-Efficacy

Reduced perception of competence and a low level of self-efficacy for being physically active were two limiting factors found in the discourses of adolescents from 12 studies e.g., [29,35,36,45,49]. Feelings of incompetence were often related to not having fun in PA, PA avoidance or peers’ teasing [29,36,49]; with a negative body image; and with the body transition during adolescence [27,36,38]. Conversely, in 15 studies, higher levels of these constructs were reported by more active adolescents e.g., [29,31,34,55] and were positively related to a positive self-concept, fun, co-participation in PA with friends, challenging activities, autonomy, and a supportive environment [27,32,34,40]. Importantly, these features seem to distinguish those adolescents who remained physically active for years from those who have abandoned PA [31,34,36].

##### Perceptions of Body Image, Femininity, and Sociocultural Norms

A negative perception of body image, exposure concerns, and the prevailing socio-cultural and religious norms related to the body image ideals and the role of girls in PA, sport, and society (e.g., PA is not for girls, is not ‘feminine’) were considered important PA obstacles found in 18 studies e.g., [32,35,38,41,47,48]. These factors affected mainly girls, especially the older ones and those from ethnic minorities. For them, social media played a negative role [44,53,56] and this should be reversed [44,56]. Regarding facilitators, having a positive body image, reduced self-presentational concerns or being active agents in resisting and challenging gender norms in PA contexts characterized those adolescents, mainly physically active girls [35,36,39,48,56]. Improving body shape, physical appearance, and weight control were also identified as reasons for PA e.g., [41,43,56].

##### Youth Agency

The opportunity for having autonomy in PA and PE, a voice, and a choice over the learning activities was identified as a PA barrier in six studies [36,40,53], and as a facilitator of PA by diverse adolescents in 15 studies [31,34,45,46,47,48,54,55].

#### 3.3.2. Theme 2: Social and Relational factors

##### Influence of Friends and Peers

The negative influence of friends on adolescents’ PA was mentioned in 21 studies and were related to lack of relationships and type of friendship groups e.g., [27,30,32]; lack of support and no co-participation in PA e.g., [30,31,38,45]; having inactive friends or that preferred other leisure activities e.g., [27,30,32,38]; doing sedentary activities with friends (9, 19); and peer pressure, teasing, and negative experiences [29,31,35,43]. Some inactive girls revealed having difficulties in dealing with peer pressure and preferred to do PA with close friends, in a single-sex and non-competitive environment e.g., [29,31,32,38]. Positive influences were identified in 27 out of 30 studies, with adolescents mentioning: Active friends that serve as role models e.g., [27,29,30,32,49,55]; friends’ presence and co-participation [30,33,37,52]; and friends’ support to start a new activity, being active, and sustaining PA involvement [27,30,32,33,34,37]. Friends were fundamental for increasing fun, learning, performance, psychosocial benefits, overcoming gender stereotypes, and other PA barriers e.g., [27,30,32,34,52,53]. Active adolescents appreciated the challenge of competing with their competent friends [29,36,37] and had the skills for dealing with time management and peer pressure, namely in later phases of adolescence. The activities adolescents engaged with were dependent on the type of friendship groups [27,30,32,36,54].

##### Influence of Family

The negative influence of family was identified in 19 studies, with adolescents—mainly those with low PA levels and girls—, referring factors such as lack of family support (financial, logistic, encouragement) e.g., [32,34,41,47,53,56]; reduced PA levels and awareness by families [31,36,41,43,48]; home-based duties and rules imposed by family increasing with age, due to safety concerns, and/or gender-related sociocultural and religious norms e.g., [41,43,47,48,56]; family pressure for academic results, sports performance, and for doing PA [44,45,53,55]. The family positive influence emerged in the discourses of adolescents across 22 studies, namely due to having a family who: provides support (encouragement, financial, transportation, first subscriptions, observation) e.g., [29,33,34,44,48,49,55]; is physically active, function as role models, did PA together and/or valued PA e.g., [30,32,36,38,43]; and provided continuous support from childhood to adolescence [34,36,49].

##### Influence of Significant Others

Coaches, PE teachers, and significant others were mentioned as barriers and facilitators to PA in 15 and 13 studies, respectively. PE teachers that provided limited support for girls, lower-skilled adolescents, and that acted as agents for promoting healthism and performative body discourses had a negative influence [34,35,44], as well as overly competitive coaches [34,49] and the presence of undesirable users [33,44,47,51]. Conversely, PE teachers [32,34,36,37,44,55] and coaches [32,34] that were supportive and enthusiastic; open to dialogue; able to establish positive relationships and sustaining a mastery-oriented motivational climate; and help in the identification of diverse PA opportunities in the community functioned as facilitators of an active lifestyle. Celebrities and popular athletes, healthcare providers, and other users were identified as possibly having a positive role in the adolescents’ PA [44,51,55,56].

#### 3.3.3. Theme 3: Physical Activity Nature Factors

##### Fun

Having fun was identified as a strong facilitator of PA involvement across 19 studies. Although the idea of fun was not the same for all adolescents, co-participation and interacting with friends was the most mentioned factor in 14 studies e.g., [32,34,38,43,45,51,54,56] followed by the type of activities. In this regard, mainly inactive girls, tended to value those activities that were: enjoyable and valued (e.g., yoga, dance, netball, tech-based fitness) [40,43,51]; focused on fun not on performance [37,52]; light intensity [40,43]; and that occurred in single-sex [52], diverse, and supportive environments [32,36,51]. Fun was also related to being active with family [32,40], feeling good and competent e.g., [34,35,38,48,54,56], having autonomy, and achieving own goals [34,38,40]. Fun was highlighted as one of the main important factors for the continuation of PA involvement [32,34,36]. In four studies, lack of fun was associated with activities that were: compulsory, repetitive, formal, and that involved traditional sports [40,54]; where the adolescents had a low perception of competence and autonomy [36,40], and reduced interactions with friends [34,36].

##### School-Based Physical Activity and Physical Education

School-based PA experiences and PE classes were identified as important factors influencing adolescent overall PA levels in 18 studies e.g., [39,42,56]. At the school level, several factors were related to limiting PA involvement, namely: no or limited PA opportunities [37,42]; traditional activities and an overly competitive environment [36,37,38,48,52]; PA not valued by school directors and members [36,41,44,55], and exclusive focus on ‘study’ [41,44]; lack of facilities, equipment [39,50,55,56], and organized PA opportunities in recess [50]. Conversely, the availability and diversity of PA opportunities in school were considered important factors [36,37,42,57]. Additionally, the school supportive overall PA culture/environment for PA was highlighted [54,55].

At the PE level, PE classes faced several obstacles related mainly to insufficient/limited infrastructures, equipment, number of teachers, and insufficient curricular time [41,53,56]. At a pedagogical level, the adolescents highlighted having many negative experiences in PE, due to bad relationships established with colleagues and teachers [35,36,41,47,49]; teachers not giving enough attention to PA [41,42], not giving students a choice [29,40,45,52]; co-educational and overly competitive environments [29,36,40]; providing activities that were non-meaningful, non-enjoyable, and that were not transferable to different contexts [40]; and exposure concerns by girls e.g., [36,38,45]. Conversely, the adolescents—mainly the actives—, identified that PE had a positive influence due to: support provided and pedagogical capacity of teachers [34,36,49]; supportive and mastery climate in PE [29,31,36,49,55]; having a voice and choice in PE, particularly girls [29,31]; enjoying PE and the activities proposed—alternative, fun, and challenging [32,38,49], which can be transferable to different life contexts [29,40,49,56]; grouping with sporty friends—active [37] or closer friends—inactive girls [36,37]; and having positive experiences in PE since the early years [36,38].

#### 3.3.4. Theme 4: Life Factors

##### Time and Competing Activities to Physical Activity

Lack of time was identified as a major PA barrier in 18 studies, particularly after the transition from childhood to adolescence e.g., [3,31,38,41,53]. Not having enough time to regularly do PA was related to the time required for studying e.g., [30,41,44,47,48,49], family duties, part-time jobs, and other conflicting obligations e.g., [48,54,56,57]. Family duties were higher for adolescent girls, namely those from ethnic minorities or that were living in lower-middle to upper-middle-income economy countries [41,43,44,48,56]. Lack of time for PA was also justified by their preference for doing other leisure activities, such as being with friends and screen-based sedentary activities e.g., [43,49,53]. Time management skills and the ability to deal with competing demands, as well as with peer and family pressures, distinguished the active from inactive adolescents [27,34,36,50,55].

##### Life-Course and Physical Activity-Related Factors

In 13 studies, adolescents identified the following specific factors for PA decline with age, particularly in the transition from primary to secondary school: decrease in perceived competence, motivation, and attitude towards PA and sport [29,37,38,49]; lack of time [27,36,39,54]; new social demands and preference for other leisure (sedentary) activities [31,41,43]; increase of study workload and pressure [27,36,39,54]; lack/reduction of support from family and/or friends [27,36,38,41]. In addition, girls identified specific challenges, such as: perceived social norms and the pressure to ‘act more girly; be less sporty and childish’ [31,43]; body changes [36,38]; and increased self-presentational concerns in PA [27,32,36,38,54]. Contrariwise, the following factors associated with a sustained PA involvement with age were identified in seven studies: positive and diverse early experiences in PA and sport [27,34,36]; favorable and ongoing social support from family, friends, and significant others [27,29,32,34,36,49]; social interaction and shared experiences of success with friends [27,32,34]; improvements in performance and valuing competition [29,32,34]; prolonged engagement in sport [34]; fun, motivation, and self-efficacy in PA [27,32,34]; challenge gender and PA social norms [27,29,36]; and time management skills [27,34,36].

#### 3.3.5. Theme 5: Sociocultural and Environmental Factors

In 25 studies, several factors related to environmental features were perceived by adolescents to be barriers to PA. These factors were related to PA programs, spaces, infrastructures, and equipment that were: distant/not accessible e.g., [33,40,41,44,48,49,52,56]); expensive e.g., [39,41,49,51,52,53,56]); unsafe [33,39,41,43,49,55,56,57]; and limited, of low quality, not specific to adolescents [43,45,50,52]. Negative weather conditions were also identified [40,44]. Furthermore, as for the PA programs, existing opportunities were: based on traditional sports [49], reduced [27,36,40,56], not specific for girls [43,45,52], non-inclusive with a preference for high-skilled adolescents [29,52], and did not meet adolescents’ preference and needs [43,49,52,54,55,56]. Girls [43,44,45,52,54,56,57] and low SES adolescents [36,43] faced further difficulties with these negative conditions. On the other hand, the following environmental facilitators were identified by adolescents (mainly active) in 17 studies: availability, accessibility (close to home, low cost), safety (well supervised, lighted), and quality (clean, well-maintenance, specific to adolescent) of spaces and PA equipment e.g., [44,48,51,52,55]); availability and accessibility of significant, organized, and other community PA programs and opportunities [33,34,45,49,50,56,57]. Additionally, as for PA opportunities, adolescents recommended activities that are: accessible, informal, unusual, diverse, significant, and enjoyable [52]; inclusive and challenging [40,44]; fun [40,51,52]; with no strict rules and affordable [51]; that they can choose [40,52]; that occur outdoor [33]; and occur in adventurous environments [51].

## 4. Discussion

This systematic review updates knowledge from qualitative research on adolescents’ perspectives on the facilitators and barriers of PA, which, in this section are organized and discussed below around the five higher-order themes to highlight important relationships within the socio-ecological model dimensions and components: (1) Individual factors (psychological—attitudes, competence, self-efficacy, body image, motivation, agency; cognitive—knowledge and understanding; physical—fitness and motor skills); (2) social and relational factors (family, friends, significant others); (3) PA nature factors (fun, school-based PA and PE; recommendations for PA); (4) life factors (time and competing activities; life-course); and (5) sociocultural and environmental factors. At the end, strengths and limitations of this study are discussed.

### 4.1. Individual Factors

An appropriate level of physical fitness and motor skills distinguished the active from the inactive adolescents [35,49,56]; as well as a heightened motivational profile, involving a positive attitude, self-concept, self-efficacy, and an intrinsic motivation e.g., [31,34]). Conversely, having an unfavorable motivational profile emerged as a major PA barrier e.g., [38,40], corroborating quantitative [6,58] and qualitative evidence [12,13,17]. Thus, teachers need to consider specific strategies for increasing adolescent’s physical fitness and motor skills which might facilitate their involvement in PA and benefit health [59,60]. This may come as a consequence of teachers nurturing adolescent motivation, self-efficacy, and attitude towards PA. Specifically, educating children for a lifetime of PA through enhancing motivation and meaning [61,62] and consequently, empowering adolescent’s basic psychological needs—autonomy, competence, and relatedness—is recommended [63,64]. Pedagogical approaches that explicitly cater for youngsters’ integrated development of literacy, competency, and enthusiasm may be adequate avenues for the promotion of children’s democratic and inclusive dispositions towards lifelong PA, irrespective of their gender, skill level or SES [65].

For adolescent girls, mainly those physically inactive, older and/or from ethnic minorities e.g., [29,41,44], a negative perception of body image, self-presentational concerns, and integration of sociocultural and religious norms related to the body image ideals and the role of girls in PA, were identified as PA barriers. Limiting conceptions about body, gender, and performativity should be challenged by teachers. Helping adolescents deconstruct those sociocultural discourses and identify the resources they can use to resist are other relevant strategies [29,31,35]. Indeed, active girls were characterized by having a better perceived body image and actively resisting and challenging these norms [29,35,48,56].

Despite adolescents identifying some PA benefits e.g., [30,34,35], their prevailing knowledge and understanding of PA, health, and fitness concepts still seems very limited e.g., [43,56] following previous evidence [66,67]. To more effectively promote PA-related knowledge, it has been recommended that teachers need to be better prepared and avoid simplistic approaches to and conceptions of PA, health, and fitness [68].

### 4.2. Social and Relational Factors

Social and relational factors identified by the studies replicate previous reviews [13,21], referring to the influence by family, friends, and significant others e.g., [27,29,32,35,37,45,52] as they interact with the adolescents’ individual factors to function as facilitators or barriers [5]. Family and friends were more often reported as facilitators, whereas significant others were more reported as barriers. This suggests that a focus on family and friends, particularly those that might carry a longstanding relationship [27], might be preferable when working with youth on identifying positive relations that may support their PA participation [17]. The opportunity to maintain and amplify those relationships through the nature of the PA experiences e.g., [31,43,52] extends the importance of such a strategy, especially when adolescents perceive that PA reduces their time to be with friends [32]. Friend’s PA levels, co-participation, and mainly support are important factors to take into consideration when intervening for promoting adolescent PA [7,69]. While friends and peers tend to appear on the facilitators’ side, the barriers tend to focus when the peers show teasing and critiquing towards girls e.g., [49,56]), and lack of support for an approach to PA participation or by promoting avoidance behaviors towards PA through an approach to sedentary activities e.g., [27].

Regarding family support, it is important to highlight that most studies reported the lack of support as a barrier and the presence of continuous support (e.g., co-participation, encouragement, attitudes, logistic) as a facilitator [30,36,41,56]. This may be more problematic when there is an active barrier from the family, particularly concerning with associated academic performance, cultural-religious traditions and minorities, and low SES e.g., [30,33,43,47]. Therefore, it may well be necessary to develop a model of parental involvement for PA in general to support interventions for families of those demographic cohorts.

Significant others as facilitators, such as national sports heroes or celebrities [56] as well as health providers [44], may present a more contemporary relevant strategy with the advent of digital influencers and social media in the promotion of PA [44,56]. Oppositely, the absence from explicit support of closer significant others, such as PE teachers, coaches, and principals, is a commonly referred social and relational barrier to PA participation e.g., [36,37,40,41,45,49,55]. Schools, as a common environment to children and youth, with great potential for PA promotion [5], have a primary responsibility in this social and health issue, especially through PE. In addition, the educational leaders, professionals, and authorities should not present as barriers to PA.

### 4.3. Physical Activity Nature Factors

The nature of PA factors reflects features of PA, namely fun, school-based PA and PE, and the explicit relevance of recommendations for PA. Most studies referred to fun as a primary feature of the PA nature working as a facilitator across contexts and demographics, which had diverse meanings [39,42,43,44]. Nonetheless, four studies have identified fun as a barrier when being absent from PA in its different forms [34,36,40,54]. While it is tempting to reduce fun to a hedonistic perspective, it is important to stress that research on fun in relation to formal PA such as sport [70] and PE [71] has shown that, as children mature to adolescence, the meaning of fun in relation to PA evolves to a desire to continuously engage with the activity, while matching an appropriate level of challenge with skill to promote enjoyment.

Many studies present school-based PA and PE as a facilitator. It is interesting to note a commonality between the girls and inactive boys’ preferences for the features of school-based PA and PE when it is fun, inclusive, diverse, involving autonomy and choice or nontraditional e.g., [52,53,55]. However, there also seems to be a commonality between active girls and active boys towards competitive activities [29,35,56] probably due to a common involvement with sports-based PA which conflicts with the experiences of those who are inactive whether they are boys or girls [36,37]. This raises the importance to consider a multivariate analysis of gender in relation to the PA participation profile when designing interventions on the types and nature of PA.

The features of school-based PA and PE were more regularly reported as barriers to PA participation, with a particular concern on the absence of PA programs [34,50] or difficulty in accessing them [42], especially in early years education from retrospective studies [36]. Barriers included elements of lack of effectiveness [42,45] or even becoming negative experiences [36,38,52]; appropriate resources or professionals [41,53,55,56]; inactive sessions [41]; developmentally inappropriate [29]; undesired performance or competitive focus e.g., [29,45,49]); irrelevant/non-meaningful learning and repetitiveness e.g., [44,49]); lack of choice/autonomy [52,54]; unsupportive or non-inclusive environment [31,43,52] and implementing a gendered curriculum [45]. When designing school-based PA and PE interventions, these are critical features to consider towards improving the interventions’ limited effectiveness in increasing PA and reducing sedentariness in key groups [15]. Future research on the impact of PE and school influence on adolescent’s lifestyle related to PA and sedentariness is needed, particularly in populations from low and middle-income economies e.g., [41,56].

For promoting PA in diverse contexts several strategies were highlighted by adolescents, namely: listen to their voice, give them a choice e.g., [34,54,55]; offer diversified, challenging appropriate, non-competitive/competitive, unstructured, unusual, meaningful, and transferable to different life contexts activities [29,36,45]; provide fun opportunities where adolescents can interact with friends in a supportive, inclusive, safe, and mastery-oriented environment e.g., [27,30,31,32,52].

### 4.4. Life Factors

Most studies framed life-course factors as a barrier, mostly related to a lack of time for PA [37,39,41,50,56] particularly in the adolescence phase. However, some adolescents reflected how time management skills were a facilitator [27], which may be connected to those more involved in competitive PA regardless of gender [29,34]. A particularly critical facilitator seems to be a sustained PA involvement [27,30,32,34,36,49]. Gavin [34] established a set of psychosocial factors as critical facilitators and barriers for such prolonged PA participation, many of which can be supported at the level of the school, as evidenced by retrospective studies e.g., [27,36,38], considering that most children will develop to adolescence throughout the schooling stage.

### 4.5. Sociocultural and Environmental Factors

Based on adolescent’s perspectives, several environmental features limited or facilitated their participation in PA e.g., [33,40,44,50,55]. In order to promote adolescent’s PA, it is important to increase the availability, accessibility, quality and meaningfulness, and safety of the PA programs, infrastructures, and equipment [44,48,51]. Thus, available opportunities should be accessible, informal, unusual, diverse, significant, and adapted to adolescents age, as concluded by Martins et al. [13]. Limited access to a safe space (e.g., traffic, presence of others) was a major concern, particularly for urban adolescents [44]. Conversely, lack of PA facilitates and of accessible opportunities (distant; no competition for girls) were particularly mentioned by rural adolescents [29,43]. Interestingly, in one study, the active adolescents either from rural or urban zones were happy with the sport and PA opportunities available [49].

### 4.6. Study Characteristics, Strengths, Limitations, and Research Recommendations

The studies included in the present review encompass adolescents with different characteristics (gender, age, SES, PA level, ethnicity), that lived in 13 countries from different continents and income economies. Thus, this review updates and expands a former one [13] since it includes adolescents from non-English speaking countries, as well as from upper-middle-income and lower-middle-income economies. Thus, the importance of some previous barriers and facilitators of PA in different geographical and cultural contexts (e.g., motivation, self-efficacy, friends support; PE) can be validated, as well as other variables more specific to the sociocultural contexts where adolescents lived. Since few studies have specifically focused on contrasting the perspectives of adolescents with diverse SES, ethnicity, urban/rural contexts, and low economy countries, in line with other suggestions [2,6,19], the need for further research at this level is reinforced. In addition to these recommendations, systematic reviews of the literature focusing on diverse populations (e.g., children, adolescents, university students) and study designs might be useful to advance knowledge in this area (e.g., [72]).

Most studies included in this review had a cross-sectional design. Only four studies had a longitudinal design, but they were all from the same author and sample, which limits the transferability of those findings and variables for different populations and contexts. Despite the fact that some of the included studies presented a retrospective approach [27,34,38], further longitudinal research focusing on the different life-course trajectories of PA is suggested [6]. PA was self-reported in 15 studies, objectively measured in one study, and not measured in 14 studies. Future research might benefit from objectively measuring adolescent’s PA levels and then listening to their voices. Even though studies included in this review were evaluated and had a medium/high reliability, these methodological procedures might improve the quality of the evidence stemming from those studies and advancing knowledge and practice in promoting PA. At the theoretical level, 13 studies have not reported the use of any theoretical model. This is also an area that can be improved in future studies. The socio-ecological model of health promotion [5] was the most used framework. Our review also used this framework and contributed to a greater understanding of the interactions across the different level factors (e.g., fun in PA and the importance of friends; PA, body image, and sociocultural norms). Finally, another important strength is that all studies included in the review were evaluated and met a minimum quality threshold. 

## 5. Conclusions

This study has built on previous qualitative reviews to update the state of the art on the facilitators and barriers for PA participation according to young people’s perspectives, by including a wider range of national contexts with diverse income levels, participant demographics, and research designs. Despite this, some limitations subsist in relation to generalizing some of the facilitators and barriers which raises the need to more regularly make explicit the theoretical frameworks, increase retrospective and longitudinal research designs, and address a more diverse contextual representation in research, while keeping a high standard of methodological quality. Still, it is important to acknowledge that the studies included in this review had a medium to high reliability.

A substantial range of facilitators and barriers are consolidated as cross-cultural at a transnational and transcontinental level, supporting their theoretical generalizability within a socio-ecological perspective of PA. Furthermore, several innovative implications for developing tailored interventions were identified and discussed aiming at contributing to the promotion of active and healthy lifestyles among adolescents. To that end, multilevel factors should be taken into account by PA professionals. Critically, interventions need to be tailored to adolescent’s characteristics, interests, and circumstances, whereby listening to their voice, PA professionals will be better positioned to be more effective in promoting PA.

## Figures and Tables

**Table 1:** Characteristics of the included studies in the systematic review.

Author (Year) [Study Ref.]	Country	Study Design	Sample Characteristics (Number of Participants, Gender, Age, Ethnicity, PA)	Data Collection and Analysis Procedures	Main Themes Identified in the Results (Authors Own Words)	Study Quality
Martins (2020) [27]	Portugal	Cross-sectional, qualitative	*n* = 16 (8 girls); Age: 17–18 yrs; SES: Low and high; ethnicity: 14 Caucasian, 2 black; PA: 8 active (4 girls), 8 inactive (4 girls)	Questionnaire (for PA also); interview (for PA also); thematic analysis	(i) PA journeys; (ii) friends provide PA benefits; (iii) friends matter in PA, but change; (iv) against all odds—rising above others.	Reliability: High; Usefulness: High
Casey (2016) [29]	Australia	Cross-sectional, qualitative	*n* = 138 girls; Age: 14–16 yrs; SES: Mixed; PA: Low active (32%); middle active (37%); high active (31%)	Interview; focus groups; PE teacher’s subjective classification of PA; narrative analysis	(i) ‘There is no I in team’: The netballers, the dancers, and me; (ii) ‘Everyone is watching, and I am just not good enough’: Power relations and perceptions of physical competence; (iii) girl’s perceptions about normalized physically active identities; (iv) power, the body, and hierarchical peer relations: Distribution in girls’ PE lessons.	Reliability: Medium; Usefulness: High
Garcia (2016) [30]	USA	Cross-sectional, mixed methods	QUAN: *n* = 152 (80 girls); * QUAL: *n* = 53 (35 girls); * Age: 16.1 ± 0.8 yrs; SES: Middle and high; Ethnicity: 80% Caucasian; PA: 53% met MVPA guidelines (80 out of 152 adolescents)	Accelerometer (for PA also); questionnaire; focus groups; statistical and content analysis	(i) Friendship groups; (ii) teams or fun; (iii) activities with friends; (iv) friend influence on PA and on-screen time; (v) PA solo or with friends.	Reliability: High; Usefulness: High
Hill (2015) [31]	Unite Kingdom	Cross-sectional, qualitative	*n* = 25 (11 girls); * *n* = 6 girls; * Age: 13–14 yrs; Ethnicity: 4 British Indian, 1 African-Asian, 1 British; PA: Active, engaged in PE and in out-of-school activities	Photographs diaries; focus groups (using photo-elicitation, interviews (for PA also); observations of PE classes; content and discourse analysis	(i) ‘They call you “man”’. Navigating gender regulation; (ii) ‘we understand each other’. Constructing femininity in girls-only spaces; (iii) ‘she’s got a life now’. Stepping away from physical activity; (iv) ‘we should play lacrosse!’ Choice about how and with whom to be active.	Reliability: Medium; Usefulness: Medium
Laird (2018) [32]	Unite Kingdom	Cross-sectional, qualitative	*n* = 18 girls; Age: 13–15 yrs; SES: Schools within catchment areas of multiple levels of deprivation; PA: Active girls	Questionnaire (for PA also); interview; content analysis (grounding theory procedures)	Participants social networks influenced different domains of their PA behavior: (i) Organized sports participation; (ii) leisure activities; (iii) active transport; and (iv) PE.	Reliability: High; Usefulness: Medium
Van Hecke (2016) [33]	Belgium	Cross-sectional, qualitative	*n* = 30 (11 girls); * *n* = N.R.; * Age: 13–15 yrs; SES: 62.5% Low	Interview (for PA also); thematic analysis	(i) Social context; (ii) modelling; (iii) social network; (iv) social trust; (v) cohesion.	Reliability: High; Usefulness: High
Gavin (2016) [34]	Canada	Cross-sectional, qualitative	*n* = 16 (8 girls); Age: 15–17 yrs; PA: Consistent involvement in PA for at least 8 years	Interview (for PA also); thematic analysis	(i) Adolescent personal considerations; (ii) school and community resources; (iii) parental support; (iv) social interaction.	Reliability: Medium; Usefulness: High
Beltrán-Carrillo (2018) [35]	Spain	Cross-sectional, qualitative	*n* = 20 (7 girls); Age: 17–18 yrs; PA: 11 physically active (4 girls), 9 physically inactive (3 girls)	Questionnaire (for PA); interview (in-depth); content analysis	(i) The influence of healthism and ideal body discourses; (ii) ideal body discourses, femininity, and barriers to sport participation; (iii) the influence of performative body discourses in sport participation; (iv) body discourses and marginalized pupils in PE.	Reliability: High; Usefulness: High
Martins (2018) [36]	Portugal	Cross-sectional, qualitative	*n* = 16 (8 girls); Age: 17–18 yrs; SES: Low and high; Ethnicity: 14 Caucasian, 2 black; PA: 8 active (4 girls), 8 inactive (4 girls)	Questionnaire (for PA also); interview (for PA also); thematic analysis	(i) Early experiences of PE at primary school; (ii) PE experiences in middle and secondary school; (iii) the role of friendly, professional, and pedagogue PE teachers; (iv) the role of friends in PE and PA; (v) the role of schools and PE conditions on students’ active lifestyles.	Reliability: High; Usefulness: High
Owen (2019) [37]	Unite Kingdom	Cross-sectional, Mixed methods	QUAN: *n* = 110 girls; Age: 14.3 ± 0.3 yrs; * QUAL: (i) 52 girls in the open-end questionnaire, (ii) 8 girls in the focus groups; SES: From a school in an area of low-deprivation; PA: Mixed, focus groups (4 girls high active, 4 girls low-to-mid active)	Questionnaire (for PA); open-ended questionnaire; focus groups; thematic analysis	Low-to-mid active girls themes: (i) Noncompetitive activities chosen as the best PA to do within the school setting; and (ii) after-school sport culture were alternatives but only for high skilled girls who could fit the social context expectations. High-active girls themes: (i) PA perceptions (the chance to work with friends and participate in competition were prime factors); (ii) PE characteristics (grouping with other sporty peers, the nature of teacher-student, and autonomy-supportive PE activities were found as fun aspects of PA participation).	Reliability: Medium; Usefulness: Medium
Knowles (2014) [38]	Unite Kingdom	Cross-sectional, qualitative	*n* = 14 girls; Age: 13.6 ± 0.3 yrs; SES: Mixed; PA: Low active	Questionnaire (for PA also); interview; narrative analysis	(i) Shaping of psychological processes through socio-cultural narratives; (ii) embodied and physical experiences within narratives; (iii) shaping psychological processes through the embodiment transition.	Reliability: Medium; Usefulness: High
Otero (2020) [39]	Colombia	Cross-sectional, qualitative	*n* = 20 (10 girls); * *n* = 18 (9 girls); * Age: 13–16 yrs; SES: Unemployment in family 10%; from low-medium income residential areas; PA: Most did PA only in PE classes	Interview; focus group; content analysis	(i) Concept and practice; (ii) facilitators; (iii) barriers.	Reliability: Medium; Usefulness: Medium
Palmer-Keenan (2019) [40]	USA	Cross-sectional, qualitative	*n* = 31 (22 girls); Age: 14–18 yrs; SES: Low-income urban communities; Ethnicity: 58% Hispanic, 23% African American, 19% mixed race; PA: Inactive	Questionnaire (for PA also); focus groups; thematic analysis	To be appealing to teens, PA had to be: (i) Fun; (ii) within their comfort zone; and (iii) promoted by ‘cool’ and relatable personalities.	Reliability: Medium; Usefulness: Medium
Baheiraei (2016) [41]	Iran	Cross-sectional, mixed methods	QUAN: *n* = 1201 (609 girls); * QUAL: *n* = 25 (10 girls); Age: 15–18 yrs; SES: Mixed; PA: Diverse levels (mainly inactive)	Questionnaire (for PA); interview (in-depth); written narrative; content analysis	(i) The inhibitory effect of the school and peers; (ii) the inhibitory effect of the family; (iii) lack of availability and the cultural barriers for the presence of girls in the community; (iv) the effect of self-feeling and self-understanding; (v) physical and mental exhaustion.	Reliability: Medium; Usefulness: Medium
Harris (2018) [42]	Unite Kingdom	Cross-sectional, qualitative	*n* = 132 (83 girls); * *n* = 83; * Age: 13–15 yrs; PA: Diverse levels	Focus groups; interview (for PA also); content analysis	(i) Issues with young people’s knowledge and understanding of health, fitness, and PA; (ii) divides between young people’s health knowledge and health behavior.	Reliability: High; Usefulness: High
Kinsman (2015) [43]	South Africa	Cross-sectional, qualitative	*n* = 51 girls; * *n* = N.R. (inferred ~24 girls, 8 girls × 3 focus groups); * Age: 13–15 yrs; SES: From one of the most marginalized rural communities in South Africa; PA: N.R.	Focus groups; thematic analysis	(i) Poverty; (ii) body image ideals; (iii) gender; (iv) parents and home life; (v) demographic factors; (vi) perceived health effects of physical activity; and (vii) human and infrastructural resources.	Reliability: High; Usefulness: High
Rajaraman (2015) [44]	India	Cross-sectional, qualitative	* *n* = 36 (18 girls); * Age: 14–15 yrs; * SES: 72% Low, 28% high; * Ethnicity: South Asian; PA: N.R.	Focus groups; thematic analysis	(i) Perceived benefits; (ii) facilitators; (iii) disadvantages; and (iv) barriers for PA.	Reliability: Medium; Usefulness: Medium
Stride (2014) [45]	Unite Kingdom	Longitudinal, qualitative	* *n* = 14 girls; * Age: 13–15 yrs; SES: Low (from an urban school in a deprived neighborhood); Ethnicity: South Asian; PA: N.R.	Observations of PE lessons; focus groups; interviews (individual and paired); thematic analysis	(i) The girls as active agents; (ii) the importance of social relations in girls’ enjoyment and involvement in PE; (iii) the PE–PA nexus.	Reliability: High; Usefulness: High
Stride (2016) [46]	Unite Kingdom	Longitudinal, qualitative	* *n* = 13 girls; * Age: 13–15 yrs; SES: Low (from an urban school in a deprived neighborhood); Ethnicity: South Asian; PA: N.R.	Observations of PE lessons; focus groups; interviews (individual and paired; for PA also); thematic analysis	(i) Contextualizing the girls’ active involvement in PA; (ii) navigating PE spaces and negotiating experiences; iii) navigating PA spaces and negotiating experiences.	Reliability: High; Usefulness: Medium
Stride (2017) [47]	Unite Kingdom	Longitudinal, qualitative	* *n* = 13 girls; * Age: 13–15 yrs; SES: Low (from an urban school in a deprived neighborhood); Ethnicity: South Asian; PA: N.R.	Observations of PE lessons; focus groups; interviews (individual and paired; for PA also); thematic analysis	(i) Family enabling PA opportunities; (ii) challenges to young women’s PA opportunities; (iii) young women actively negotiating their physicality.	Reliability: High; Usefulness: Medium
Stride (2018) [48]	Unite Kingdom	Longitudinal, qualitative	* *n* = 13 girls; * Age: 13–15 yrs; SES: Low (from an urban school in a deprived neighborhood); Ethnicity: South Asian; PA: N.R.	Observations of PE lessons; focus groups; interviews (individual and paired; for PA also); thematic analysis	(i) PA in and around the home; (ii) ‘fragility’ and household responsibilities; and (iii) ‘fragility’, education, and schooling careers.	Reliability: High; Usefulness: Medium
Devís-Devís (2015) [49]	Spain	Cross-sectional, qualitative	*n* = 20 (7 girls); Age: 17–18 yrs; PA: Physically active and inactive	Questionnaire (for PA); interview (in-depth); content analysis	(i) Perceived (in)competence, obesity, and peer teasing; (ii) family, friends, and significant others; (iii) new social demands and preferences; (iv) physical education, knowledge, and its role in daily life; (v) urban and rural places of residence.	Reliability: High; Usefulness: High
Hannus (2018) [50]	Estonia	Cross-sectional, qualitative	*n* = 92 (56 girls); * *n* = 33; * Age: 14–16 yrs; PA: N.R.	Focus groups; thematic analysis	(i) Organized activities; (ii) PA facilities; (iii) play equipment; (iv) time, rules, and regulations; (v) unsuitable weather; (vi) experiential attitudes; (vii) instrumental attitudes; (viii) injunctive norm; (ix) descriptive norm.	Reliability: Medium; Usefulness: Medium
Hidding (2018) [51]	Netherlands	Cross-sectional, mixed methods	*n* = 115 (42 girls); Age: 13–17 yrs; SES: Schools from a low and high tercile; PA: N.R.	Concept mapping group sessions; hierarchical cluster analysis and researchers’ interpretation	Potential determinants of an activity-friendly environment belonging to four domains: (i) Physical; (ii) social; (iii) economic; (iv) motivational characteristics.	Reliability: Medium; Usefulness: High
James (2018) [52]	Unite Kingdom	Cross-sectional, qualitative	*n* = 78 (gender N.R.); Age: 13–14 yrs; PA: N.R.	Focus groups; thematic analysis	(i) Lower/remove the cost of activities without sacrificing the quality; (ii) make physical activity opportunities more locally accessible; (iii) improve the standards of existing facilities; (iv) make activities more specific to teenagers; (v) give teenagers a choice of activities/increase variety of activity and (vi) provide activities that teenage girls enjoy; (vii) increased opportunity to participate in an unstructured activity.	Reliability: Medium; Usefulness: High
Fernandez-Prieto (2019) [53]	Spain	Cross-sectional, qualitative	*n* = 25 (12 girls); Age: 13–17 yrs; SES: School from a poor zone; Ethnicity: 8 Caucasian, 3 Spanish, 2 Chinese, 2 Moroccan, 1 Russian; PA: N.R.	Focus groups; thematic analysis	(i) Motivation; (ii) barriers.	Reliability: Medium; Usefulness: Medium
Fernandez-Prieto (2019) [54]	Spain	Cross-sectional, qualitative	Photo elicitation: *n* = 26 (13 girls); focus groups: *n* = 10 (6 girls); Age: 14–16 yrs; SES: Mixed (mainly low-middle); Ethnicity: 18 Caucasian, 5 Asian; 3 Arabic, 10 Latin; PA: N.R.	Photograph elicitation; focus groups; interpretative phenomenological analysis	Photo elicitation: (i) People; (ii) space; (iii) places; (iv) components; (v) sports; (vi) food; (vii) barriers; (viii) attitudes; (ix) classification PA; (x) association PA. Focus groups: (i) Barriers; (ii) motivation; (iii) classification PA; (iv) body image and gender.	Reliability: Medium; Usefulness: Medium
Borhani (2017) [55]	Iran	Cross-sectional, qualitative	*n* = 48 girls; Age: 15–18 yrs; SES: Mixed; PA: N.R.	Focus groups; interview (in-depth); content analysis	(i) Perceived benefits; (ii) perceived barriers; (iii) perceived self-efficacy; (iv) feelings related to PA behavior; (v) interpersonal influencers; (vi) situational influencers.	Reliability: Medium; Usefulness: Medium
Abdelghaffar (2019) [56]	Morocco	Cross-sectional, qualitative	*n* = 56 (28 girls); Age: 14–16 yrs; SES: 95% Middle income; PA: N.R.	Focus groups; thematic analysis	(i) Perceived motivation and limiting factors; (ii) PA awareness; (iii) time constrains; (iv) social support; (v) gender and cultural norms; (vi) access to opportunities.	Reliability: High; Usefulness: Medium
Payán (2019) [57]	USA	Cross-sectional, qualitative	*n* = 64 (43 girls); Age: 14–18 yrs; SES: Schools located in three zones with poverty rate of 23.9%; Ethnicity: 58% Hispanic, 23% African American, 3.2% multi-ethnic, 1.6% White, 1.6% Hawaiian	Focus groups; inductive analysis (grounding theory procedures)	(i) Availability of physical activity opportunities (at school and community); (ii) interpersonal barriers (lack of motivation and of time); (iii) interpersonal facilitator (social support).	Reliability: Medium; Usefulness: Medium

Legend: MVPA: Moderate-to-vigorous physical activity; N.R: Not reported; PA: Physical activity; PE: Physical education; QUAN: Quantitative study; QUAL: Qualitative study; SES: Socioeconomic status. * Characteristics of the adolescents who met the eligibility criteria of the present systematic review.

**Table 2:** Themes and sub-themes synthesizing the main facilitators and barriers to physical activity.

Themes	Sub-Themes	Study Reference Number																														*n*	
		27	29	30	31	32	33	34	35	36	37	38	39	40	41	42	43	44	45	46	47	48	49	50	51	52	53	54	55	56	57	○	●
Individual factors	Physical and motor skills	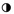	●					●	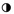	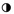	●	●		●	●	●		●					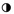				○		●	●		5	14
	PA attitude, knowledge, and understanding	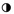	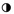	○	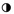			○	○	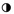	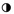	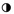	○	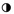	●	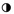	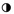	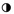	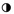	●	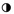	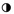	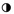		○	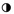	●	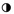	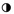	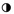		23	21
	Motivation	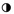			○	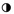		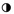		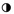	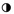	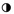	○			●	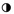	●							○	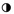	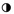	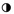	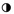	●	●	14	15
	Perception of competence and self-efficacy	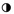	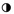		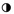	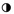		○	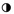	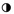	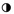	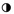		○					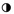	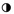			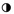				○		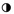			15	12
	Perceptions of body image, femininity, and sociocultural norms	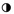	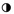		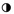	●			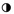	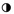		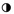	○		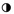	○	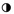	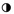	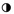	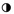	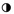	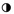				●	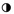	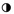	○	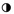		19	18
	Youth agency	○	●		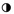			○		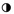	○	○		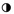					○	○	○	○				○	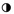	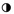	○			15	6
Social and relational factors	Friends and peers influence	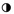	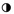	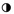	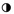	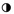	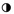	○	●	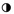	○	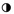	○	○	●		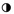	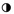	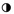	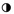	○	○	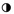	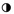	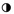	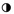	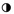	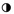	○	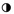	○	27	21
	Family influence	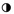	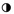	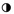		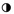	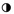	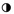		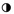		○	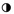	○	●		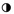	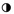	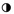	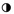	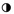	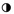	○			●	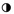	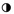	○	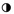	○	22	19
	Significant others influence		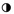			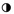	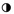	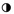	●	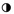	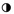			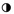				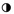	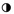	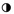			●		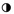				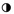	○	●	13	15
PA nature factors	Fun	○				○		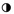	○	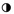	○	○	○	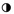		○	○	○	○			○			○	○		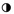	○	○		19	4
	School-based PA and PE		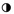		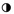	○		○	●	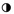	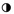	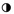	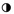	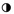	●	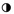		●	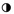	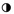		●	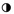	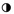		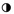	●	●	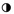	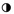	○	18	21
Life factors	Time and competing activities to PA	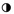			●	●		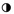		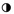	●		●		●			●	●			●	●	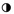			●	●	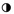	●	●	5	18
	Life-course factors	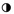	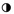		●	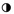		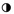		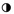	●	●	●		●		●						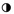					●				6	13
Sociocultural and environmental factors	Environmental factors	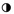	●				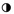	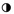	●	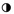	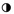	●	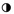	●	●	●	●	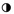		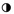		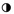	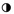	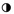	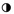	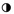	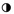	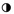	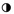	●	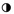	17	25

○ Facilitator; ● barrier; 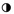 facilitator and barrier. Study reference number, first author and year of publication: 27. Martins (2020), 29. Casey (2016), 30. Garcia (2016), 31. Hill (2015), 32. Laird (2018), 33. Van Hecke (2016), 34. Gavin (2016), 35. Beltrán-Carrillo (2018), 36. Martins (2018), 37. Owen (2019), 38. Knowles (2014), 39. Otero (2020), 40. Palmer-Keenan (2019), 41. Baheiraei (2016), 42. Harris (2018), 43. Kinsman (2015), 44. Rajaraman (2015), 45. Stride (2014), 46. Stride (2016), 47. Stride (2017), 48. Stride (2018), 49. Devís-Devís (2015), 50. Hannus (2018), 51. Hidding (2018), 52. James (2018), 53. Fernandez-Prieto (2019), 54. Fernandez-Prieto (2019), 55. Borhani (2017), 56. Abdelghaffar (2019), 57. Payán (2019).

## Supplementary Materials

The following are available online at https://www.mdpi.com/article/10.3390/ijerph18094954/s1. Table S1: Inductive facilitators and barriers of physical activity; Table S2: Aims and theoretical frameworks of the studies included in the review.
