# Supplementary material for: Adolescents’ Perspectives on the Barriers and Facilitators of Physical Activity: An Updated Systematic Review of Qualitative Studies
Source: Int J Environ Res Public Health. 2021 May 6;18(9):4954. doi: 10.3390/ijerph18094954 (PMC8125166; doi:10.3390/ijerph18094954)
Supplement: Supplementary file 1 [file ijerph-18-04954-s001.zip › supplementary material_S1_proofreaded.pdf]

Supplementary material

Table S1. Inductive facilitators and barriers of physical activity

Author (year) [Study ref.]	Facilitators	Barriers
Martins (2020) [27]	1. Diverse and positive PE/PA experiences since early years with friends involved<br>2. Establishment of friendships in early years and continued relationships influenced PA later on<br>3. Friends support for PA (active, encouragement)<br>4. Meaningful interactions with friends in diverse PA contexts (having fun together, motivation)<br>5. Family support for PA<br>6. Ability to challenge gender and performance stereotypes with support from close friends (girls)<br>7. Time management skills	1. Negative experiences in PA since early years<br>2. Lack of time<br>3. Competing leisure sedentary activities<br>4. Limited PA attitude<br>5. Sociocultural norms limiting girls' PA<br>6. Lack of support from friends for PA<br>7. Peer influence on leisure sedentary activities<br>8. Peer victimization episodes in PE and PA<br>9. Lack of motivation for PA<br>10. Lack of support from family (inactive)<br>11. Limitations to access sport clubs (low SES)
Casey (2016) [29]	1. Peer and friends support<br>2. Perception of competence<br>3. Self-efficacy<br>4. Competition (active girls)<br>5. Sport/PE focus on performance (active girls)<br>6. Co-educational PE (active girls)<br>7. High SES<br>8. Family support<br>9. Being active was linked to ideals of femininity	1. Reduced perception of competence<br>2. Reduced perception of body image<br>3. Fears of being watched by peers (low/medium active girls)<br>4. Narrowed conceptions of 'ideal' bodies<br>5. Lack of peer, friends and family support<br>6. Reduced self-efficacy<br>7. Lack of appropriate PA opportunities (high skills required)<br>8. Co-educational PE (low-medium active girls)<br>9. Sport and PE focus on performance (low /medium active girls)<br>10. Prevailing gendered and performative discourses limit girls PA involvement
Garcia (2016) [30]	1. Type of friendship groups (from sport teams)<br>2. Friends and family support<br>3. Competition/competing with friends (boys)<br>4. Close friend and socializing in PA (girls)<br>5. PA benefits: health/competition (boys), psychosocial (girls)	1. Type of friendship groups (from school)<br>2. Sedentary activities with friends<br>3. Lack of PA support from friends and family<br>4. Family pressure/demanding girls to be active

Hill (2015) [31]	1. Sport skills<br>2. Motivation<br>3. Self-confidence<br>4. Carefree attitude and challenge gender norms<br>5. Perception of body image<br>6. Perception of competence<br>7. Choice in PE: meaningful/new activities, friends to play with<br>8. Safe and supportive PE environment	1. Teasing and lack of support from boys in PE<br>2. Prevailing discourses on body (fat), femininity and performance (competition, types of activities)<br>3. PE as a non-caring and non-supportive space<br>4. Co-educational PE/PA contexts<br>5. Boring and repetitive activities in PE<br>6. Lack of support from friends<br>7. Competing leisure time activities<br>8. Cultural and religious norms influence PA
Laird (2018) [32]	1. Provision of social support (from family, friends, PE teacher, coaches) lead to improvements on: (1.1.) performance, (1.2.) self-efficacy, (1.3) fun, (1.4) motivation and (1.5) enabling PA<br>2. PA modelling<br>3.Connectedness<br>4. PE positive and environment (teacher, friends)	1. Low self-efficacy for PA<br>2. Feeling self-conscious about being active<br>3. Low social support (friends, family, teachers)<br>4. Laziness and lack of motivation<br>6. Connectedness negative impact (reduced time spent with friends outside of PA)
Van Hecke (2016) [33]	1. Accessibility by foot/bicycle/public transport<br>2. Located close to home/school<br>3. Presence of active friends and family (siblings)<br>4. Cleanliness and upkeep of space/facilities<br>5. Availability of sport fields / large open spaces<br>6. Safety and aesthetics of spaces / facilities	1. Presence of undesirable users<br>2. The behaviors of others<br>3. Unsafety and lack of cleanliness<br>4. Lack of friends support and presence<br>6. Parents restriction
Gavin (2016) [34]	1. PA attitude (passion for the activity)<br>2. PA benefits (psychosocial, health)<br>3. Fun<br>4. Motivation: intrinsic, extrinsic and mastery; self-efficacy for PA<br>5. Competition and prolonged engagement in PA<br>6. Friends support to PA and social interaction<br>7. Supportive teachers/coaches; Continuos family spport for PA<br>8. Access to PA facilities/organized programs<br>9. Continuous family support for PA	1. Lack of fun<br>2. Lack of motivation<br>2. Fewer opportunities of PA with age<br>3. Injuries<br>4. Lack of time and competing leisure demands<br>5. Lack of PA facilities/programs/opportunities<br>6. Coaches: overly competitive<br>7. Lack of family support to PA

Beltrán-Carrillo (2018) [35]	1. Improve body shape through PA<br>2. Improve health (indirectly)<br>3. Competition (active boys and girls)<br>4. Fun and positive PA attitude<br>5. Good physical fitness and sport skills<br>6. Perception of competence	1. Fear of masculinized female bodies<br>2. Non-hegemonic body shape (not "fit") and peer teasing<br>3. Ideals of femininity limit types of PA practiced<br>4. Low perception of competence and sport skills<br>5. PE teachers favor students with fit bodies<br>6. Performative discourses (competition, victory)
Martins (2018) [36]	1. Diverse, positive PE/PA experiences since early years<br>2. Positive PE/PA attitude and fun<br>3. Motivation (mastery orientation)<br>4. Availability PA opportunities in the community<br>5. Perception of competence<br>6. Activities in PE: diversified, unusual, challenging; competitive (boys), inclusive and mixed gender (active girls), multi-activities, choice<br>7. PE teacher pedagogical capacity<br>8. Friends support in PA and PE<br>9. PE and school role in promoting PA<br>10. Family support for PA (continuous)<br>11. Good sport skills	1. Limited out-of-school formal PA activities<br>2. Limited access to PE in early years (low SES)<br>3. Early negative PE and PA experiences<br>3. Lack of motivation, fun and laziness<br>4. Limited physical fitness and sports skills<br>6. Reduced perception of competence<br>7. Body image and self-presentational concerns (girls)<br>8. Limited PE/PA attitude<br>9. Competition and high-intensity activities in PE (inactive boys)<br>10. PE teacher lack of support<br>11. Limited PE and school role in promoting PA<br>12. Lack of support from friends and peer teasing<br>14. Lack of support from family for PA
Owen (2019) [37]	1. Enjoyment of PA (fun, benefits of PA)<br>2. Doing PA while having fun with friends<br>3. Noncompetitive activities (inactive girls)<br>4. 'Fun' sessions to encourage more girls to practice (inactive girls)<br>5. Competition (active girls)<br>6. Supportive teachers and autonomy in PE/PA<br>7. Grouping with sporty friends in PE/PA (active girls) and compete with friends (active)<br>8. Motivation: focus on personal improvement<br>9. After-school sport clubs: increase participation and retention (active girls)<br>10. Perception of competence	1. Limited knowledge/understanding of PA<br>2. Lack of time<br>3. Lack of motivation<br>4. Lack of PA options<br>5. Not sporty or low sport skills<br>6. Pressure to win and to compete (inactive girls)<br>7. After school club culture (for athletes only)<br>8. Lack of teachers support for low skilled girls<br>9. Lack of fun (low and middle active girls)<br>10. Reduced perception of competence

Knowles (2014) [38]	1. Perception of competence<br>2. Fun<br>3. PE and other organized activities (primary school phase)<br>4. Support from family<br>5. Support from friends<br>6. Motivation<br>7. Active lifestyle self-identity<br>8. PA attitude<br>9. PE attitude<br>10. No self-presentational concerns	1. Secondary school phase transition<br>2. PE promotes “active identities”, contradicting the “socially acceptable feminine identity”<br>3. Unfavorable perception of body image<br>4. Self-presentational concerns<br>5. Reduced perceived competence<br>6. Preference for other leisure sedentary activities<br>7. Limited PA attitude and lack of motivation<br>8. Acceptance for specific sport decrease with age<br>9. Lack of friends support<br>10. Physical pain associated with specific PA<br>11. Dominant cultural values on PA, gender and performance shape psychological processes
Otero (2020) [39]	1. PA benefits (health, body image, weight management, socialization)<br>2. Family support to PA (same sex parent)<br>3. Motivation<br>4. Fun<br>5. Availability/access of PA facilities and opportunities in the community<br>6. Activities: non-structured, open air<br>7. PE classes as a mean to promote PA<br>8. Co-participation in PA with friends	1. Lack of time<br>2. School study and homework commitments<br>3. Lack of family support<br>4. Sport spaces at the school<br>5. Lack of s2port spaces at the neighborhood<br>6. Cost of existing PA opportunities<br>7. Unsafe to do PA in the neighborhood<br>8. Lack of access to school sport facilities
Palmer-Keenan (2019) [40]	1. Fun activities: technology-based fitness, free and accessible online workouts, doable at home<br>2. Acceptable sports: low intensity<br>3. Alternative activities offered in PE<br>4. Inclusive and easy challenging activities<br>5. Comfort (activities, context) and choice<br>6. Support from family and friends<br>7."Promoter" characteristics: cool, relatable, good looking, celebrity, youtuber, athlete<br>8. Perception of competence and self-efficacy	1. Lack of accessible low-cost PA opportunities<br>2. Preference for leisure sedentary activities<br>3. Not fun: sports and formal exercise, PE, jogging<br>4. Do not promote PA by emphasizing the health benefits of PA<br>5. Physical discomfort: sweat, tired, body hurts<br>6. Being active outdoors during adverse weather conditions<br>7. No messages from directors/parents/authority<br>8. Lack of alternative activities and choice in PE

Baheiraei (2016) [41]		1. Studying-based schools' environment and school directors not giving importance to PE and PA<br>2. PE: inactive classes, negative experiences, lack of teachers and of equipment<br>3. Lack of friends and family support<br>4. Lack of weight management and body image<br>5. Time constraints<br>6. Lack of available, accessible and safe spaces<br>7. Social and cultural norms limit PA (girls)<br>8. Low self-esteem and mental exhaustion<br>9. Physical weakness and poor fitness
Harris (2018) [42]	1. PA benefits physical health<br>2. PA benefits appearance, weight, shape, size<br>3. Fun<br>4. Schools and PE teachers are promoting healthy and active lifestyles<br>5. Autonomy and choice in PE	1. Limited understanding of PA-health benefits<br>2. Conceptual confusion of PA, fitness and health<br>3. Do not know recommendations of PA for health<br>4. Laziness, tiredness and illness<br>5. Lack of motivation<br>6. Bad weather and no easy access to PA programs<br>7. School and PE can improve their role in promoting active and healthy lifestyles
Kinsman (2015) [43]	1. Body image ideals (pressure to be thin)<br>2. Available facilities and competitions for boys<br>3. Family support<br>4. Benefits of PA for health<br>5. Gender (boys)<br>6. Age (younger adolescents)<br>7. Fun and motivation<br>8. PA to make friends and socialize	1. Culturally embedded body image ideals<br>2. Criticism / teasing from friends and peers<br>3. Lack of facilities and competitions for girls<br>4. Lack of family support<br>5. Unsafe environment<br>6. Gender (girls), age (older) and poverty<br>7. Competing leisure sedentary activities<br>8. Nationality and lack of legal documentation<br>9. Lack of understanding of PA benefits<br>10. Extrinsic motivation<br>11. Culturally embedded disincentives for girls' PA

Rajaraman (2015) [44]	1. PA benefits: health, academic performance, physical fitness, socialization, ideal body shape<br>2. Social support from friends, family and teachers<br>3. Social support from significant others (health providers)<br>4. Social media messages and role modeling<br>5. Accessibility of playgrounds<br>6. Fun	1. Limited understanding of PA-health benefits<br>2. Perceived PA disadvantages<br>3. Lack of friends, family, teachers and significant others support (girls)<br>4. Weather and pollution (urban)<br>5. Academic pressure and reduced PA (in school)<br>6. Health problems / overweight and obesity<br>7. Fatigue, body pain, injuries<br>8. Low perception of body image (girls)<br>9. Lack of motivation (girls)<br>10. Reduced sport opportunities for girls; limited access to safe space/facilities (urban)<br>11. Societal norms related to girls in sport<br>12. Lack of time
Stride (2014) [45]	1. Perception of competence<br>2. Fun<br>3. Play and learn with your friends<br>4. Activities: indoor, new to everyone<br>5. Attitude towards PE<br>6. Perception of body image<br>7. Single sex-PE classes<br>8. Being active agents resisting dominant discourses<br>9. Multiple identities and disruption of traditional gender codes	1. Lack of perception of competence<br>2. Lack of friends and family support<br>3. PE activities: outside, competition, repetitive, irrelevant, gendered curriculum<br>4. Lack of available/accessible sport clubs/spaces<br>5. Study pressure and family-imposed duties<br>6. Exposure concerns and embarrassment in PE<br>7. Cultural/religious norms related to girls in sport<br>8. Negative PE and PA attitude<br>9. PE teacher opinions of girl's abilities/pedagogies<br>10. PE limited role in promoting PA outside school
Stride (2016) [46]	1. Family support in PA<br>2. Friends support in PE<br>3. Available/accessible/safe spaces for PA/sport<br>4. Single sex-PA contexts<br>5. Competition/performance in PE (some girls)<br>6. Perception of competence	1. Negative PE and PA attitude<br>2. Activities in PE: irrelevant, repetitive, obligatory<br>3. Peers teasing in PE<br>4. Low perception of competence<br>5. Exposure concerns and embarrassment in PE<br>6. Lack of family support<br>7. Cultural/religious norms related to girls in PA<br>8. Formal, organized and Eurocentric spaces
Stride (2017) [47]	1. Family support in PA<br>2. Girls has active agents negotiating their physicality and different cultural challenges	1. Family lack of support for PA (specific activities, gender and religious related obstacles).<br>2. Cultural/religious norms related to girls in PA<br>3. Negative PE and PA attitude

Stride (2018) [48]	1. Available, accessible, safe PA spaces near home<br>2. PA at home (private/safe space, degree control)<br>3. Fun<br>4. PA benefits: health, stress relief<br>5. Body weight management<br>6. Girls as active agents negotiate their physicality<br>7. Family and friends' social support	1. Lack of parents support<br>2. Household responsibilities<br>3. Belief systems/environments within different PA/sport contexts that challenge their right to embody their religion and be physically active<br>4. School and PE focus on the "physical performance and sport agenda"
Devís-Devís (2015) [49]	1. High perception of competence<br>2. Family support (in childhood and adolescence)<br>3. Friends support to PA<br>4. PA benefits: relieve stress<br>5. Applying knowledge acquired in PE to out-of-school PA (active)<br>6. PE teachers support for starting to do PA/sport<br>7. Available and significant sport opportunities<br>8. Physical fitness and sport skills (active)	1. Low perception of competence<br>2. Negative attitude towards PA<br>3. Lack of time<br>4. Peer teasing (low skilled, girls, obese)<br>5. Coaches/PE teachers' behaviors and negative experiences<br>6. "Sportivization" process of PE and its contents<br>7. Learning came must from sport not PE (active), learning in PE not significant<br>8. Lack of available/significant sport opportunities
Hannus (2018) [50]	1. Availability of PA facilities, equipment and organized PA opportunities<br>2. Sufficiency of time<br>3. Be with friends	1. Lack of PA facilities, equipment, organized PA opportunities<br>2. Behavioral and normative beliefs<br>3. Insufficiency of time and time regulations
Hidding (2018) [51]	1. Physical characteristics: clean, well-maintained, proximity, suitable<br>2. Social characteristics: Ambience (fun, cozy), being allowed to be active (no strict rules), presence of others (positive)<br>3. Economic characteristics: Affordable.<br>4. Motivational characteristics: Challenging, motivating, adventurous environment	1. Presence of others (negative)<br>2. The need to bring additional equipment to a sports facility

James (2018) [52]	1. Reduced cost of activities<br>2. Quality of facilities (safe, clean, lights)<br>3. Improve local access to PA opportunities<br>4. Activities: specific to adolescents, informal, diversified, non-conventional, non-competitive; that adolescents enjoy<br>5. Give adolescents a choice/voice<br>6. Provide activities that girls enjoy and fun<br>8. Opportunity to be active with friends, enjoy and socialize (girls)<br>9. Inclusion<br>10. Motivation (intrinsic)	1. Being red and sweaty in front of boys (girls)<br>2. Lack of family support<br>3. High cost of activities<br>4. Low quality of facilities and sports equipment; Lack of local facilities need to travel to venues<br>6. Activity provision that does not include/encourages/motivates adolescents<br>7. Age restrictions to participate in activities<br>8. Not giving adolescents a choice/voice<br>9. PE: activities that are repetitive, traditional, structured, for adults; negative experiences<br>10. Non-autonomous motivation
Fernandez-Prieto (2019) [53]	1. Available, accessible, safe and aesthetic spaces<br>2. Motivation<br>3. Satisfaction with the performance of PA<br>4. Friends co-participation in PA and fun<br>6. Support from family<br>7. Support from school<br>8. Perception of body image (girls)	1. Lack of motivation and fun<br>2. Fatigue and laziness<br>3. Lack of support from friends and family<br>4. PE: compulsory; boring and repetitive activities<br>5. Lack of time<br>6. Low attitude towards PA<br>7. Lack of available/accessible /safe spaces (girls)<br>8. Inappropriate use of the woman figure in sport by the social media (girls)<br>9. Social and cultural norms limit PA (girls)<br>10. Negative perception of body image (girls)
Fernandez-Prieto (2019) [54]	1. Motivation<br>2. Social support from friends and family<br>3. Physical fitness<br>4. Activities: team sports, competition, new, chosen by adolescents<br>5. Available spaces for PA in the neighborhood<br>6. Neighborhood multiculturalism and new forms of PA<br>7. Perception of body image (girls)	1. Lack of time<br>2. Competing leisure sedentary activities<br>3. Lack of motivation<br>4. Low PA attitude<br>5. Low perception of body image (girls)<br>6. PE classes (limited time)<br>7. Lack of support from family (girls) and friends<br>8. Economic cost to access sport facilities<br>9. Activities: imposed, repetitive, no autonomy<br>10. Social media do not value girl's PA<br>11. Cultural norms limit PA (girls)
Borhani (2017) [55]	1. Perceived benefits of PA (health, improvement appearance, fitness, socialization, sleep)<br>2. Self-efficacy and self-regulation<br>3. Positive feelings related to PA (motivation, fun)<br>4. Support from family, friends, PE teachers, significant others (popular athletes)<br>5. Supportive school PA and PE environment	1. PA induced fatigue and injuries<br>2. Low self-efficacy and motivation for PA<br>3. Lack of sport skills to exercise<br>4. Lack of planning ability for PA (knowledge)<br>5. Lack of time<br>6. Competing leisure sedentary activities<br>7. Study workload and pressure

	6. Available/safe spaces and sport equipment<br>7. Ability to choose any favorite sport<br>8. Ability to use information to learn new PA<br>9. Time management skills<br>10. Cultural attitudes towards girls in PA	8. Lack of available/accessible/safe spaces for PA<br>9. Negative feelings related to PA<br>10. School: directors do not give importance to PE, inadequate facilities for PA
Abdelghaffar (2019) [56]	1. Fun<br>2. Competition (boys)<br>3. Benefits of PA: health<br>4. Perception of body image<br>5. Weight control<br>6. PA awareness and positive attitude<br>7. Support from family<br>8. Support from friends<br>9. Significant others: role models by national sports celebrities<br>10. PE: contributes to improve overall PA levels	1. Physical disability, poor fitness, low sport skills<br>2. Laziness (no motivation)<br>3. PA lack of awareness and negative attitude<br>4. Time constraints<br>5. Lack of support from family and friends<br>6. Fear of being intimidated (girls) and fear of injuries<br>7. Shame to do PA outside (girls)<br>8. Cultural norms limit PA (girls)<br>9. PE: lack of time, infrastructures, equipment<br>10. School: policy relate to PE and sport spaces<br>11. Lack of available/accessible/safe spaces (girls)
Payán (2019) [57]	1. Availability of PA opportunities in school and community<br>2. Social support from friends<br>3. Social support from family	1. Lack of motivation and laziness<br>2. Lack of time<br>3. Unsafety<br>4. Unwanted attention and threats (girls)
