# Supplementary material for: Adolescents’ Perspectives on the Barriers and Facilitators of Physical Activity: An Updated Systematic Review of Qualitative Studies
Source: Int J Environ Res Public Health. 2021 May 6;18(9):4954. doi: 10.3390/ijerph18094954 (PMC8125166; doi:10.3390/ijerph18094954)
Supplement: Supplementary file 1 [file ijerph-18-04954-s001.zip › supplementary material_S2_proofreaded.pdf]

Supplementary material

Table S2. Aims and theoretical frameworks of the studies included in the review

Author (Year) [Study ref.]	Aims (author's own words)	Theoretical framework
Martins (2020) [27]	To explore the experiences and perspectives of physically active and inactive adolescents regarding the influence of their friends on their PA throughout childhood and adolescence	Social-ecological model of health
Casey (2016) [29]	To explore the discourses and power relations operative between groups of girls that appeared to influence their participation in PE and outside of school in sport and PA in rural and regional communities	Foucault's techniques of power
Garcia (2016) [30]	To determine the association between adolescent MVPA and screen time with their nominated friends' behaviors and explore potential mechanisms of friends' social influences on MVPA and screen time	Not reported
Hill (2015) [31]	To share girls' perspectives on how the discourses of femininity and body in PE affected their gender performances and activity engagement	Not reported
Laird (2018) [32]	To identify how girls perceive different sources and types of support to influence their PA, and to develop a grounded theory of how social support influences PA in adolescent girls	Social support
Van Hecke (2016) [33]	To determine which social and physical environmental factors affect adolescents' visitation and PA in POS in low-income neighborhoods	Social-ecological model of health
Gavin (2016) [34]	To explore adolescents' perceptions of psychosocial factors influencing their prolonged involvement in sports and PA	Social cognitive theory and health action process approach
Beltrán-Carrillo (2018) [35]	To analyze how socio-cultural discourses about the body influenced adolescents' participation in PA	Body discourses and embodied identities
Martins (2018) [36]	To explore and analyse the perspectives of physically active and inactive adolescent boys and girls with different socioeconomic status, regarding the meanings that PE had on their physical activity lifestyles throughout childhood and adolescence	Socio-ecological model for PE
Owen (2019) [37]	To explore adolescent girls' perceptions and experiences of school-based PA to inform a subsequent intervention, the Girls Peer Activity project	Not reported
Knowles (2014) [38]	Understand the decrease in PA behavior in adolescent girls during the transition from primary to secondary school in relation to their socio-cultural and embodied/physical experiences	Not reported
Otero (2020) [39]	To describe the perception of adolescents about the facilitators and barriers that influence PA	Not reported
Palmer-Keenan (2019) [40]	To ascertain inactive teens' insights regarding the types of physical activities they would be willing to do, and to inform a education - PA social marketing campaign targeting this audience	Not reported
Baheiraei (2016) [41]	To explain adolescents' insufficient PA and its related factors	Not reported
Harris (2018) [42]	To explore young people's knowledge and understanding of health, fitness and PA	Not reported
Kinsman (2015) [43]	To identify and describe facilitating factors and barriers that are associated with PA among adolescent girls in rural, north-eastern South Africa, and to develop a model for promoting leisure-time PA within this population	Cultural competence and social identity theory

Rajaraman (2015) [44]	To understand perceived benefits, facilitators, disadvantages, and barriers for physical activity among South Asian adolescents in India and Canada	Not reported
Stride (2014) [45]	To explore what South Asian Muslim girls have to say about school-based PE and how this relates to their involvement in PA away from school	Hill Collins' matrix of domination and intersectionality
Stride (2016) [46]	To explore the kinds of settings in which girls undertake PE and PA, and how these spaces influence experience	Hill Collins' matrix of domination and intersectionality
Stride (2017) [47]	To understand and explore South Asian Muslim young women's experiences of PA and how these are influenced by family	Hill Collins' matrix of domination and intersectionality
Stride (2018) [48]	To explore the PA experiences in and around home of South Asian, Muslim young women	Hill Collins' matrix of domination and intersectionality
Devís-Devís (2015) [49]	To explore the socio-ecological factors and their interplay that emerge from a qualitative study and influence adolescents' PA and sport participation	Social-ecological model of health
Hannus (2017) [50]	To explore the factors that perceivably affect students' recess PA	Theory of planned behavior and the social-ecological model of health
Hidding (2018) [51]	To explore adolescents' perspectives on characteristics of an activity-friendly environment through concept mapping (N.R.)	Not reported
James (2018) [52]	To explore the recommendations made by teenagers to improve activity provision, uptake and sustainability of PA engagement for both themselves and their peers	Not reported
Fernandez-Prieto (2019) [53]	To identify adolescents' perceived barriers and motivations to regular PA practice, and how they form the concept of PA through thought and action	Not reported
Fernandez-Prieto (2019) [54]	To explore the topics related to PA of a group of adolescents and to identify the barriers and motivators of regular PA practice	Not reported
Borhani (2017) [55]	To explore teenage girls' experiences regarding the determinants of PA promotion	Pender's Health Promotion Model
Abdelghaffar (2019) [56]	To explore potential social-ecological barriers and facilitators of PA in Moroccan adolescents	Social-ecological model of health
Payán (2019) [57]	To understand perceived barriers and facilitators to PA among at-risk African American and Hispanic adolescents and adults in a low-income community	Social-ecological model of health
